# Characteristics of Women Enrolled in a Patient Portal Intervention for Menopause

**DOI:** 10.1089/whr.2020.0091

**Published:** 2020-10-26

**Authors:** Avi H. Lamba, Kiranmayee Muralidhar, Anika Jain, Fei Tang, Orlando Gomez-Marin, Silvina Levis, Stuti Dang

**Affiliations:** ^1^ Flint Hill School, Oakton, Virginia, USA.; ^2^ Department of Public Health, and University of Miami Miller School of Medicine, Miami, Florida, USA.; ^3^ Research Service, Miami Veterans Affairs Healthcare System, Miami, Florida, USA.; ^4^ Department of Medicine, University of Miami Miller School of Medicine, Miami, Florida, USA.; ^5^ Geriatric Research, Education and Clinical Center (11 GRC), Miami Veterans Affairs Healthcare System, Miami, Florida, USA.

**Keywords:** menopausal symptoms, menopause, patient portal, shared decision-making

## Abstract

***Background:*** We developed a 6-month educational intervention addressing menopause and management of menopausal symptoms called “My Health*e*Vet to Enable And Negotiate for Shared decision-making” or MEANS. MEANS is offered through secure messaging *via* the My Health*e*Vet patient portal system.

***Materials and Methods:*** Women veterans aged 45–60 years registered at the Miami, West Palm Beach, and Orlando Veterans Affairs Healthcare Systems (VAHS). Intervention group: women in the Miami VAHS enrolled in My Health*e*Vet who were sent an invitation, agreed to participate, and completed the baseline survey. Comparison group: women from the Miami, West Palm Beach, and Orlando VAHS who responded to the baseline survey.

***Results:*** The intervention group enrolled 269 women at Miami VAHS: average age 53.2 years; 42.4% white, 43.1% black, and 24.2% Hispanic; 95.9% already used My Healthe Vet. The Comparison group had 590 women: average age 53.8 years; 70.8% white, 20.7% black, and 10.2% Hispanic; 57.6% already used My Healthe Vet.

***Conclusions:*** The differences between the intervention and comparison groups likely represent the regional demographic variations and the disparate recruitment techniques adopted for the two groups. Using within- and between-group comparisons at the end of the 6-month intervention, this novel project will evaluate the feasibility of a patient portal intervention on knowledge and shared decision-making regarding menopause among racially and ethnically diverse women. The study highlights the scalable and enormous potential for patient portals in nonurgent chronic disease management and shared decision-making, important in the existing health care climate, wherein “meaningful use” of electronic health records is mandated. Because of the COVID-19 pandemic, medical care has abruptly changed to telehealth and this approach to patient education is more relevant now than ever before.

This quality improvement project's registration number is ClinicalTrials.gov ID: NCT03109145.

## Introduction

In North America, there are ∼30 million women in the menopausal age range between 40 and 54 years^1^ and roughly 6,000 women reach menopause daily.^2^ Menopause is defined as the permanent cessation of menstrual periods. The time after reproductive years and before menopause is known as the menopausal transition or perimenopause. Perimenopause and menopause disrupt the lives of many women. Common symptoms include hot flashes, night sweats, insomnia, mood instability, and vaginal dryness, which have a negative effect on the quality of life.^3,4^ A majority of health care providers do not screen to identify symptomatic menopausal women and also fail to address and treat menopausal symptoms in their patients.^5^ Women undergoing this change have expressed a desire for up-to-date information about menopause and how to cope with menopausal symptoms.^1^ Women in this age group often have difficulty scheduling health care visits due to conflicting work or family responsibilities.^6^ Thus, there is a need for developing innovative educational programs on menopause.^5^

Patient portals are secure online websites that give patients convenient 24-hour access to personal health information.^7^ The Department of Veterans Affairs (VA) has its own robust patient portal, My Health*e*Vet. Besides allowing veterans to securely access their medical records online, My Health*e*Vet gives them the ability to communicate with their health care team using its secure messaging (SM) function. To use My Health*e*Vet, veterans register online, and they have to “opt in” to use SM. Patients can also schedule appointments and request prescription refills on My Health*e*Vet.^8^ Patient portals offer a promising approach to improve patient knowledge and outcomes in their chronic conditions such as heart failure^9^ and diabetes.^10^ However, the use of patient portals for menopause and to improve shared decision-making is yet to be explored.^11,12^

Shared decision-making involves sharing the best available evidence between patients and providers and supporting patients in the process of weighing options and making informed health or treatment choices.^13^ The shared decision-making process is facilitated by patients' understanding of their diagnosis and available treatment options.

The “My Health*e*Vet to Enable And Negotiate for Shared decision-making” (MEANS) is a pilot educational intervention about the management of menopause for women veterans using My Health*e*Vet.^14^ In this study, we describe the patient recruitment and baseline characteristics of MEANS participants.

## Methods and Materials

The guiding framework for this study is a modified “Three Talk Model of Shared Decision Making for Clinical Practice.”^13^ The Three Talk model breaks down the shared decision-making process into three practical steps: Step 1 called Team Talk engages the patients and initiates a conversation, making sure that patients know that a decision needs to be made and reasonable options are available; Step 2 called Option Talk provides patients the best available evidence. This is often best done outside the clinic visit encounter since patients want time to study new information, consider their personal preferences, and discuss with others. Step 3 called Decision Talk allows the patient and provider to make a shared decision regarding the best option by considering the different options and preferences, during the clinical encounter. The shared decision-making process provides patients with the support they need to make the best individualized care decisions.

**FIG. 1.**
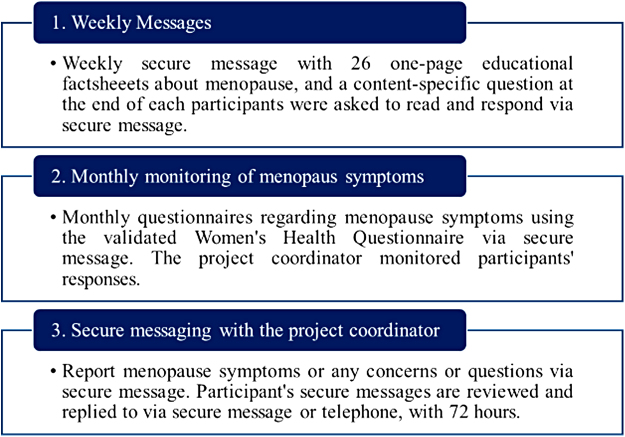
Menopause intervention delivered *via* My Health*e*Vet.

In this study, we leverage the VA's patient portal, My Health*e*Vet. Since shared decision-making can be facilitated by preparatory education, our intervention largely focuses on educating patients regarding menopause. We designed educational activities to provide patients the best evidence about available options (Fig. 1**)**, intending to enable a shared decision together with their provider during the face-to-face clinical encounter.^15^

MEANS is an unblinded nonrandomized pilot educational intervention for women in the Veterans Affairs Healthcare System (VAHS). The MEANS project involved an intervention and a comparison group. We evaluate the impact of a 6-month educational intervention delivered *via* My Health*e*Vet on self-reported knowledge of menopause. Secondary outcomes include patient perception of shared decision-making and rates of diagnosis and medication management of menopause and its symptoms. We collect information from the participants regarding menopause knowledge, menopause symptoms, shared decision-making regarding menopause, and satisfaction with the MEANS intervention after 6 months. A broad overview of the schedule is shown in Table 1.

**Table 1.** Objectives, Outcome Measures, and Schedule for Intervention and Comparison Groups

		Collection schedule	
Objective	Measure	Baseline	6 Months
Both intervention and comparison groups			
Primary objective: knowledge			
Menopause knowledge	Validated knowledge questionnaire and rating of self-perception of menopause knowledge	X	X
Secondary objective: shared decision-making			
Shared decision-making	Overall and menopause-specific occurrence	X	X
Rate of menopause diagnosis and medication management of menopause	No. of women with menopause diagnosis, menopause symptom type, frequency, and treatment for menopause symptoms	X	X
Program evaluation—intervention group only			
Usability of My Health*e*Vet and secure messaging	Ease of use of My Health*e*Vet and secure messaging	X	X
	Frequency of use of My Health*e*Vet and secure messaging	X	X
	Barriers to the use of My Health*e*Vet		X
Program satisfaction	Satisfaction with the intervention		X

The study was reviewed and approved by the Miami VAHS Institutional Review Board and determined to not require informed consent since it is a quality improvement educational intervention.

The MEANS project involved identifying female veterans of perimenopausal and menopausal age at three VAHS. The MEANS intervention was offered only at the Miami VAHS, whereas the comparison group included women at the Miami, West Palm Beach, and Orlando VAHS.

### Recruitment methods

#### Inclusion criteria

Participants were women aged 45–60 years who had at least one primary care visit during the previous year at the West Palm Beach, Orlando, or Miami VAHS.

#### Intervention group recruitment methods

Eligibility criteria included having at least one primary care visit during the previous year at the Miami VAHS, already registered for My Health*e*Vet and SM, or willing to do so with the help of study staff. Study recruitment included sending introductory information *via* SM describing the intervention to eligible women in the Miami VAHS. We asked these women to contact us by SM or by phone if they were interested. We also contacted by telephone some women who had used My Health*e*Vet but had not opted in for SM. They were explained the project, offered instructions and assistance with registering for My Health*e*Vet, and in the use of SM by the project coordinator. We asked these women to complete a baseline survey. Women who agreed to participate in the MEANS intervention comprised the intervention group.

#### Comparison group recruitment methods

Using electronic medical record data, we identified all eligible women from the West Palm Beach and Orlando VAHS, and sent them the baseline surveys *via* mail, along with a stamped return envelope. Those who responded were made part of the comparison group, which also included women from the Miami VAHS who were not willing to participate in the intervention but completed the baseline survey.

### Statistical analysis

Descriptive statistics was used to assess the distributional properties of the different variables and their interrelationships, as well as to determine missing data and detect outliers. Baseline characteristics were presented as frequency (percentage) for categorical variables, and as mean ± standard deviation (SD) for continuous variables. The one-way analysis of variance (ANOVA) was used to compare the means of the continuous variables between the intervention and comparison groups. Chi square tests were used for the comparison of independent proportions. Paired *t*-tests were used for the analysis of pre- and postcontinuous data. Logistic regression was used to study the association between SM use and age. Results with a *p*-value of <0.05 were considered statistically significant. All analyses were performed using SPSS 25.0 for Windows (SPSS, Inc., Chicago, Illinois) or R version 3.4.4 (R Foundation for Statistical Computing).

## Results

### Participant recruitment

#### Intervention group recruitment

**FIG. 2.**
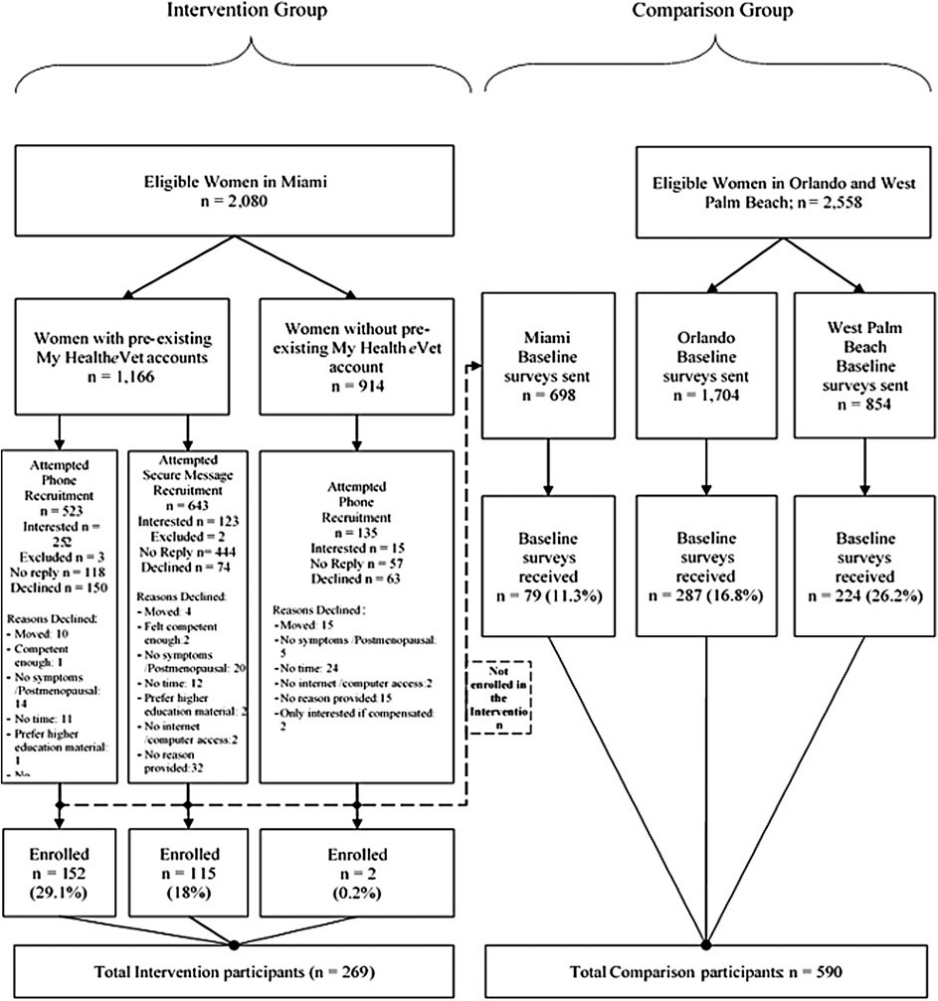
Enrollment in the MEANS project by site. MEANS, My Health*e*Vet to Enable And Negotiate for Shared decision-making.

In the Miami VAHS, using data from the electronic medical records, 2,080 women veterans were identified as potential participants, of which 1,166 (56%) had My Health*e*Vet and were contacted. Of the 643 women who were contacted through SM, we enrolled 115 (18%) in the project. Among the 523 women who we contacted by phone, 152 (29%) enrolled. Among the eligible women identified in Miami VAHS who did not have My Health*e*Vet and SM (*n* = 914), we enrolled two women of the 135 we contacted in the study. In total, we enrolled 269 women at the Miami site in the intervention group. Figure 2 shows enrollment in the MEANS project by group.

The most common reasons women declined the intervention were as follows: they moved to a different city (29 women), felt competent enough (3), had no symptoms/postmenopausal (39), had no time (47), preferred higher education material (3), had no Internet/computer access (7), provided no reason (157), and were only interested if compensated (2).

#### Comparison group recruitment

We sent a total of 854 surveys at the West Palm Beach VAHS and received 224 (26.2%) back. We sent a total of 1,704 surveys at the Orlando VAHS and received 287 (16.8%) back. Of 698 surveys for the Miami VAHS, we received 79 (11.3%) responses. The response rate was significantly different by site (*p* < 0.01).

In total, 590 baseline surveys were returned, as shown in Figure 2.

### Participant baseline characteristics

#### Demographics

Table 2 provides baseline characteristics of the 269 intervention and 590 comparison group participants by site. At baseline, the mean ages of the two groups were similar, at 53.2 years (SD = 4.4) for the intervention group and 53.8 years (SD = 4.5) for the comparison group. However, the women in the intervention group in Miami had a greater percentage of younger women between the ages of 45 and 50 years (32.3%), compared with the comparison group (25.1%) (*p* = 0.04). The Miami intervention group had significantly more Hispanic participants (24.2%) compared with the overall comparison group (10.2%) (*p* < 0.01). Approximately 43.1% of participants in the intervention group were African American, compared with 20.7% in the comparison group (*p* < 0.01), and 42.4% of participants in the intervention group were white, compared with 70.8% in the comparison group (*p* < 0.01). Intervention and comparison groups also differed by marital status (*p* ≤ 0.01), level of education (*p* = 0.01), and employment (*p* < 0.05).

**Table 2.** Demographic Characteristics of the Participants

Characteristics, N (%)	All sites (N = 859)	Intervention group Miami (N = 269)	Miami comparison (N = 79)	Orlando comparison (N = 287)	West Palm comparison (N = 224)	All comparison (N = 590)
Age						
45–50^*^	235 (27.4)	87 (32.3)	21 (26.6)	77 (26.8)	50 (22.3)	148 (25.1)
51–55	285 (33.2)	89 (33.1)	21 (26.6)	99 (34.5)	76 (33.9)	196 (33.2)
56–61	330 (38.4)	92 (34.2)	35 (44.3)	108 (37.6)	95 (42.4)	238 (40.3)
N/A	9 (1.0)	1 (0.4)	2 (2.5)	3 (1.0)	3 (1.3)	8 (1.4)
Mean age	53.6	53.2	54.1	53.5	54.1	53.8
Ethnicity^**^ (Hispanic)						
Yes	125 (14.6)	65 (24.2)	20 (25.3)	19 (6.6)	21 (9.4)	60 (10.2)
No	721 (83.9)	201 (74.7)	56 (70.9)	266 (92.7)	198 (88.4)	520 (88.1)
N/A	13 (1.5)	3 (1.1)	3 (3.8)	2 (0.7)	5 (2.2)	10 (1.7)
Marital status						
Married/marriage like relation^**^	381 (44.4)	101 (37.5)	30 (38.0)	140 (48.8)	110 (49.1)	280 (47.5)
Divorced	326 (38.0)	115 (42.8)	29 (36.7)	99 (34.5)	83 (37.1)	211 (35.8)
Widowed	50 (5.8)	10 (3.7)	6 (7.6)	22 (7.7)	12 (5.4)	40 (6.8)
Never married^*^	91 (10.6)	39 (14.5)	12 (15.2)	24 (8.4)	16 (7.1)	52 (8.8)
N/A	11 (1.3)	4 (1.5)	2 (2.5)	2 (2.9)	3 (1.3)	7 (1.7)
Race						
Asian	6 (0.7)	2 (0.7)	1 (1.3)	1 (0.3)	2 (0.9)	4 (0.7)
Black or African American^**^	238 (27.7)	116 (43.1)	22 (27.8)	62 (21.6)	38 (17.0)	122 (20.7)
American Indian or Alaskans	7 (0.8)	3 (1.1)	1 (1.3)	2 (0.7)	1 (0.4)	4 (0.7)
Native Hawaiian or Pacific islanders	4 (0.5)	1 (0.4)	0 (0)	0 (0)	3 (1.3)	3 (0.5)
White^**^	532 (61.9)	114 (42.4)	40 (50.6)	208 (72.5)	170 (75.9)	418 (70.8)
Others^**^	52 (6.1)	23 (8.6)	14 (17.7)	8 (2.8)	7 (3.1)	29 (4.9)
N/A	20 (2.3)	10 (3.7)	1 (1.3)	6 (2.1)	3 (1.3)	10 (1.7)
Level of education						
No high school diploma	5 (0.6)	0 (0.0)	0 (0)	3 (1.0)	2 (0.9)	5 (0.8)
High school graduate^**^	70 (8.1)	17 (6.3)	4 (5.1)	18 (6.3)	31 (13.8)	53 (9.0)
Some College, no degree	270 (31.4)	77 (28.6)	29 (36.7)	89 (31.0)	75 (33.5)	193 (32.7)
College degree or higher^*^	505 (58.8)	170 (63.2)	45 (57.0)	176 (61.3)	114 (50.9)	335 (56.8)
N/A	9 (1.0)	5 (1.9)	1 (1.3)	1 (0.3)	2 (0.9)	4 (0.7)
Employment status						
Employed	505 (58.8)	149 (55.4)	47 (59.5)	170 (59.2)	139 (62.1)	356 (60.3)
Unemployed	115 (13.4)	35 (13.0)	6 (7.6)	39 (13.6)	35 (15.6)	80 (13.6)
Disabled^**^	121 (14.1)	49 (18.2)	19 (24.1)	29 (10.1)	24 (10.7)	72 (12.2)
Retired^*^	105 (12.2)	28 (10.4)	6 (7.6)	48 (16.7)	23 (10.3)	77 (13.1)
N/A	13 (1.5)	8 (3.0)	1 (1.3)	1 (0.3)	3 (1.3)	5 (0.8)
Annual income ($)						
<10,000	45 (5.2)	9 (3.3)	3 (3.8)	16 (5.6)	17 (7.6)	36 (6.1)
10,001–19,999	81 (9.4)	32 (11.9)	7 (8.9)	22 (7.7)	20 (8.9)	49 (8.3)
20,000–29,999	90 (10.5)	27 (10.0)	4 (5.1)	29 (10.1)	30 (13.4)	63 (10.7)
30,000–39,999	114 (13.3)	32 (11.9)	17 (21.5)	43 (15.0)	22 (9.8)	82 (13.9)
40,000–49,999	82 (9.5)	27 (10.0)	5 (6.3)	22 (7.7)	28 (12.5)	55 (9.3)
50,000–59,999	74 (8.6)	22 (8.2)	6 (7.6)	23 (8.0)	23 (10.3)	52 (8.8)
>60,000	252 (29.3)	83 (30.9)	22 (27.8)	91 (31.7)	56 (25.0)	169 (28.6)
Prefer not to respond	107 (12.5)	32 (11.9)	12 (15.2)	39 (13.6)	24 (10.7)	75 (12.7)
N/A	14 (1.6)	5 (1.9)	3 (3.8)	2 (0.7)	4 (1.8)	9 (1.5)

All the statistically significant differences are indicated by the symbols ^*^ or ^**^. These *p*-values reflect the difference between the intervention and all comparison groups: ^*^ reflecting a *p*-value <0.05, and ^**^ reflecting a *p*-value <0.01.

#### Literacy and use of computers and My Health*e*Vet

Both intervention and comparison groups were similar regarding health literacy and numeracy, and most participants in both groups previously used computers and the Internet (Table 3).

**Table 3.** Comparison of Health Literacy, Numeracy, and Computer Use by Group and Site

Question,* N *(%)	All sites (*N* = 859)	Intervention group Miami (*N* = 269)	Miami comparison (*N* = 79)	Orlando comparison (*N* = 287)	West Palm comparison (*N* = 224)	All comparison (*N* = 590)
Health literacy						
How confident are you filling out medical forms by yourself?						
All of the time	664 (77.3)	208 (77.3)	58 (73.4)	220 (76.7)	178 (79.5)	456 (77.3)
Most of the time	124 (14.4)	39 (14.5)	11 (13.9)	46 (16.0)	28 (12.5)	46 (16.0)
Some of the time	42 (4.9)	14 (5.2)	8 (10.1)	12 (4.2)	8 (3.6)	28 (4.7)
A little of the time	10 (1.2)	3 (1.1)	0 (0.0)	4 (1.4)	3 (1.3)	7 (1.2)
None of the time	7 (0.8)	0 (0.0)	1 (1.2)	3 (1.0)	3 (1.3)	7 (1.2)
Numeracy						
How good are you at working with fractions?						
Extremely good	261 (30.4)	87 (32.3)	21 (26.6)	88 (30.7)	65 (29.0)	174 (29.5)
4	249 (29.0)	70 (26.0)	25 (31.6)	86 (30.0)	68 (30.4)	179 (30.3)
3	200 (23.3)	69 (25.7)	21 (26.6)	64 (22.3)	46 (20.5)	91 (15.4)
2	83 (9.7)	26 (9.7)	6 (7.6)	30 (10.5)	21 (9.4)	57 (9.7)
Not good at all	54 (6.3)	11 (4.1)	5 (6.3)	17 (5.9)	21 (9.4)	43 (7.1)
How good are you at figuring out how much a shirt will cost if it is 25% off?						
Extremely good	479 (55.8)	143 (53.2)	36 (45.6)	171 (59.2)	129 (57.6)	336 (56.9)
4	202 (23.5)	69 (25.7)	24 (30.4)	60 (20.9)	49 21.9)	133 (22.5)
3	96 (11.2)	30 (11.2)	12 (15.2)	28 (9.7)	26 (11.6)	66 (11.2)
2	45 (5.2)	17 (6.3)	4 (5.1)	17 (5.9)	7 (3.1)	28 (3.2)
Not good at all	27 (3.1)	8 (3.0)	2 (2.5)	8 (2.8)	9 (4.0)	19 (3.2)
How often do you find numerical information to be useful?						
Very often	414 (48.2)	130 (48.3)	37 (46.8)	136 (47.4)	111 (50.0)	284 (48.1)
4	230 (26.8)	79 (29.4)	21 (26.6)	82 (28.6)	49 (21.9)	152 (25.8)
3	144 (16.8)	39 (14.5)	11 (13.9)	48 (16.7)	45 (20.1)	104 (17.6)
2	46 (5.4)	14 (5.2)	8 (10.1)	12 (4.2)	12 (5.4)	32 (5.4)
Never	14 (1.6)	4 (1.5)	1 (1.2)	7 (2.4)	2 (0.9)	10 (1.7)
Computer use						
Ever used computer?						
Yes	845 (98.4)	265 (98.5)	76 (96.2)	285 (99.3)	219 (97.7)	580 (98.3)
No	3 (0.4)	0 (0)	1 (1.3)	0 (0)	2 (0.9)	3 (0.5)
N/A	11 (1.3)	4 (1.5)	2 (2.5)	2 (0.7)	3 (1.3)	7 (1.2)
Ever used Internet?						
Yes	846 (98.5)	265 (98.5)	77 (97.5)	285 (99.3)	219 (97.7)	581 (98.5)
No	4 (0.5)	0 (0)	1 (1.3)	0 (0)	3 (1.3)	4 (0.7)
N/A	9 (1.05)	4 (1.5)	1 (1.3)	2 (0.7)	2 (0.9)	5 (0.8)
Ever used search engines?^*^						
Yes	826 (96.2)	262 (97.4)	72 (91.2)	280 (97.5)	212 (94.6)	564 (95.6)
No	22 (2.5)	4 (1.5)	5 (6.3)	4 (1.4)	9 (4.01)	18 (3.1)
N/A	11 (1.28)	3 (1.1)	2 (2.5)	3 (1.05)	3 (1.3)	8 (1.4)

All the statistically significant differences are indicated by the symbols ^*^ or ^**^. These *p*-values reflect the difference between the intervention and all comparison groups: ^*^ reflecting a *p*-value <0.05 and ^**^ reflecting a *p*-value <0.01.

The intervention and comparison groups were also assessed at baseline on their familiarity and use of the My Health*e*Vet patient portal (Table 4). Participants in the intervention group were significantly more likely to use My Health*e*Vet more frequently. Among the participants in the intervention group, only 4.1% reported never using My Health*e*Vet, compared with 42.4% (*p* < 0.01) in the comparison group. The intervention group participants reported significantly higher use of all My Health*e*Vet functions compared with the comparison group (*p* < 0.01). The intervention and comparison groups differed by their perceived difficulty in using My Health*e*Vet, with 71.4% of the intervention group participants ranking the My Health*e*Vet system as easy, whereas only 29.2% of the comparison group gave it the same score (*p* < 0.01).

**Table 4.** My Health*e*Vet and Secure Messaging Use by Group and Site

My Healthe*Vet use, *N (%)	All participants (N = 859)	Intervention group Miami (N = 269)	Miami comparison N = 79	Orlando comparison (N = 287)	West Palm comparison (N = 224)	All comparison (N = 590)
How frequently have you used My Health*e*Vet in past 30 days?						
Daily	14 (1.6)	9 (3.3)	1 (1.3)	3 (1.05)	1 (0.5)	5 (0.8)
Few times/week^**^	56 (6.5)	33 (12.3)	6 (7.6)	12 (4.2)	5 (2.2)	23 (3.9)
Once a week^**^	66 (7.6)	42 (15.6)	6 (7.6)	8 (2.8)	10 (4.4)	24 (4.1)
Every 2–3 weeks^**^	138 (16.1)	68 (25.3)	18 (22.8)	34 (11.8)	18 (8.03)	70 (11.9)
Once	131 (15.3)	40 (14.9)	16 (20.3)	37 (12.9)	38 (16.9)	91 (15.4)
Did not use in past 30 days	179 (20.8)	64 (23.8)	19 (24.05)	55 (19.2)	41 (18.3)	115 (19.5)
Never used^**^	261 (30.3)	11 (4.1)	6 (7.6)	137 (47.7)	107 (47.7)	250 (42.4)
N/A^**^	14 (1.6)	2 (0.7)	7 (8.9)	1 (0.3)	4 (1.8)	12 (2.0)
Functions used on My Health*e*Vet (answered yes)^**^						
Secure messaging	348 (40.5)	197 (73.2)	35 (44.3)	64 (22.3)	52 (23.2)	151 (25.6)
Meds renewal	354 (41.2)	186 (69.1)	45 (56.9)	68 (23.7)	55 (24.5)	168 (28.5)
Check appointments	316 (36.8)	173 (64.3)	37 (46.8)	61 (21.3)	45 (20.08)	143 (24.2)
Make appointments	94 (10.9)	64 (23.8)	8 (10.1)	7 (2.4)	15 (6.7)	30 (5.1)
Check laboratories	273 (31.7)	150 (55.8)	26 (32.9)	50 (17.4)	47 (20.9)	123 (20.8)
Read health information	229 (26.6)	136 (50.6)	21 (26.6)	46 (16.02)	26 (11.6)	93 (15.8)
Print health records	91 (10.5)	58 (21.6)	11 (13.9)	6 (2.09)	16 (7.1)	33 (5.6)
Other	134 (15.6)	26 (9.7)	5 (6.3)	63 (21.9)	40 (17.9)	108 (18.3)
Difficulty level with using My Health*e*Vet						
Easy^**^	380 (44.2)	192 (71.4)	39 (49.3)	85 (29.7)	64 (28.6)	188 (31.8)
Neither easy nor hard	112 (13)	36 (13.4)	17 (21.5)	30 (10.5)	29 (12.9)	76 (12.9)
Hard	80 (9.3)	21 (7.8)	10 (12.6)	27 (9.4)	22 (9.8)	59 (10)
N/A^**^	287 (33.4)	20 (7.4)	13 (16.5)	145 (50.5)	109 (48.6)	267 (45.3)
Would you use My Health*e*Vet to converse with VA team?^**^						
Already using	238 (27.7)^**a**^	149 (55.4)	24 (30.3)	42 (14.6)	23 (10.3)	89 (15.1)
Yes	411 (47.8)^**a**^	95 (35.3)	45 (56.9)	150 (52.3)	121 (54.02)	316 (53.6)
No	114 (13.3)	14 (5.2)	8 (10.1)	52 (18.1)	40 (17.9)	100 (16.9)
N/A	96 (11.2)	11 (4.1)	2 (2.5)	43 (14.9)	40 (17.9)	85 (14.4)
Have you ever consulted VA for menopause symptom management?						
Yes^**^	325 (37.8)	136 (50.6)	33 (41.7)	80 (27.8)	76 (33.9)	189 (32.0)
No^**^	490 (57)	125 (46.5)	45 (56.9)	191 (66.5)	129 (57.6)	365 (61.9)
N/A^*^	44 (5.1)	8 (3.0)	1 (1.3)	16 (5.6)	19 (8.5)	36 (6.1)
Have you ever used secure messaging to contact VA team for menopause symptom management?						
Yes	109 (12.6)	45 (16.7)	17 (21.5)	24 (8.3)	23 (10.2)	64 (10.9)
No^*^	678 (78.9)	212 (78.8)	53 (67.1)	240 (83.6)	173 (77.2)	466 (79.0)
N/A^*^	72 (8.3)	12 (4.5)	9 (11.4)	23 (8.01)	28 (12.5)	60 (10.2)
How often would you use secure messaging in future for menopause symptom management?						
More than once a week^*^	55 (6.4)	26 (9.7)^b^	7 (8.8)	11 (3.8)	11 (4.9)	29 (4.9)^b^
More than once a month^**^	146 (16.9)	74 (27.5)^b^	15 (18.9)	34 (11.8)	23 (10.3)	74 (12.2)^b^
Once a month or less	338 (39.3)	111 (41.3)^b^	27 (34.2)	117 (40.7)	83 (37.05)	227 (38.5)^b^
Never^**^	256 (29.8)^**c**^	46 (17.1)	27 (34.2)	100 (34.8)	83 (37.05)	210 (35.6)
N/A^*^	64 (7.5)	12 (4.5)	3 (3.8)	25 (8.7)	24 (10.7)	52 (8.8)

All the statistically significant differences are indicated by the symbols ^*^ or ^**^. These *p*-values reflect the difference between the intervention and all comparison groups: ^*^ reflecting a *p*-value <0.05 and ^**^ reflecting a *p*-value <0.01.

^a^ Respondents willing to use My Health*e*Vet to converse with their VA team.

^b^ Respondents willing to use secure messaging in the future for menopause management.

^c^ Respondents stating they are not willing to use secure messaging in the future.

VA, Veterans Affairs.

Among the patients who were registered for My HealtheVet, we also analyzed for patient engagement with SM related to age. Logistic regression was used to study the association between SM use and age. The results showed that age was not associated with being an SM user (*p* = 0.64). In addition, we noticed that there was no difference in the mean age for SM users who had used it at least once in the past 30 days (53.4 ± 4.4) and nonusers who had not used SM in the past 30 days or had never used it (53.6 ± 4.6) (*p* = 0.64). We also looked at the association between the frequency of SM use and age group (45–49, 50–54, 55–59, and ≥59 years) among the SM users, and age groups were not associated with the frequency of SM usage (chi square test, *p* = 0.36).

There were differences between the intervention and comparison groups in women who would be willing to use My Health*e*Vet to communicate with their VA provider teams. At baseline, 55.4% of participants in the intervention group were already using My Health*e*Vet to communicate with their providers, compared with 15.1% of individuals in the comparison group (*p* < 0.01). In addition, 35.3% of the intervention group participants and 53.6% of the comparison group participants expressed willingness to communicate with the VA provider team *via* SM (*p* < 0.01).

#### Participant use of My Health*e*Vet to discuss menopausal symptom management

In the intervention group, 50.6% of participants had consulted the VA for menopausal symptom management (*p* < 0.01), compared with 32% of participants in the comparison group; however, the majority of participants in both groups had never used SM to discuss menopausal symptom management. The largest percentage of participants in both groups (41.3% in the intervention group and 38.5% of the comparison group) chose an SM frequency of once a month or less frequently to discuss menopausal symptom management. Few participants reported that they would never use SM to discuss menopausal symptom management, including 17.1% of the intervention group and 35.6% of the comparison group (*p* < 0.01).

#### Miami intervention and comparison

Taking into account only the groups from Miami, we found that 73.2% of the intervention group use the SM function compared with 44.3% of the comparison group (*p* < 0.01). Among the intervention group, 71.4% of women found it easy, 7.8% found it hard, and 13.4% said it was neither easy nor hard to use My Health*e*Vet. Among the Miami comparison group 49.3% of women found it easy, 12.6% found it hard, and 21.5% said it was neither easy nor hard to use My Health*e*Vet (*p* < 0.01). Although 55.4% of the intervention group and 30.3% of the comparison group said they were already using My Health*e*Vet to converse with the VA team (*p* < 0.01), 35.3% of the intervention group and 56.9% of the comparison group said they would be willing to do so in the future (*p* < 0.01).

## Discussion

Our patient portal project for menopause education successfully recruited 269 women for the intervention from one VA medical facility, representing 13% of the eligible women of that age group at that medical center. This group had an average age of 53 years; 42% were white, 43% black, and 25% Hispanic; and 95.7% had previously used My Health*e*Vet. The comparison group comprised 590 women from three VA facilities. More than 98% in both groups previously used a computer, Internet, and search engines. Women in both groups were similar in average age, health literacy and numeracy, income, and previous use of computers and the Internet. However, the intervention group had more of the following types of women: younger (between 45 and 50 years), with a college degree or higher, blacks, Hispanics, divorced or never married, and My Health*e*Vet users who used most functions, including more frequent SM.

Given the distinctive characteristics of the general population in Miami compared with those in West Palm Beach and Orlando, it is unsurprising that our intervention and comparison groups differed in ethnicity. Our data reflect the U.S. Census data for 2018, showing comparable ethnic differences in these cities with 71.2% of Miami's population being Hispanic or Latino compared with 22.8% in West Palm Beach, and 29.2% in Orlando. The intervention and comparison groups also differed in racial demographics, matching data from previous reports that minorities are more likely to report using SM compared with nonminorities.^16,17^

As of 2014, Pew Research data show that 81% of U.S. adults used computers at work, home, school, or elsewhere, 90% had a cell phone, and 58% had a smartphone.^18^ Furthermore, nearly three-fourth of Internet users and more than half of smartphone owners looked online for health or medical information,^19^ indicating that online health portals could be a good source of health education. In a study examining veterans and military personnel specifically, almost all active service members, guard/reserve members, and veterans in the study used computers. In addition, three-fourth reported willingness to download a health-related application to their technology devices.^19^ Similarly, our study demonstrates that most veteran women in the 45–60 years age group have used a computer and the Internet. Given their familiarity, these women should also be able to navigate the My Health*e*Vet portal.

However, our recruitment experience shows that having computer access and using the Internet are not sufficient to enroll patients in technology interventions, but rather an engagement of participation with the specific technology, in this case, the My Health*e*Vet portal, is required. Previous studies have confirmed that portal usage is associated not just with a computer and broadband access and usage, but with overall online behavior as well.^11,20,21^ Although almost all women in Miami reported having and using computers, the recruitment yield was really low unless they were already registered for My Health*e*Vet, and “opted in” to use SM. In addition, despite already being registered for My Health*e*Vet, several women declined participation in the study citing several other reasons (moved to a new city, had no Internet access at the time, preferred higher education material, felt competent enough, had no time, were asymptomatic, or were only interested if they were compensated). This highlights the challenge of reaching patients who are not already engaged with similar technology, yet offers an opportunity for targeting and training the nonusers to improve recruitment.^22^ In addition, our data show that even among the secure message users, there was a higher percentage of women who agreed to participate when approached by telephone rather than by SM. This is probably related to the human interaction versus computer only in this group. This is an area that needs more research and integration into today's health care, especially with increasing use of patient portals.

In response to the question, “Would you use My Health*e*Vet to converse with the VA team?” about a quarter of the overall respondents said they were already using My Health*e*Vet to consult the VA, and almost half of them said they would be willing to use My Health*e*Vet to communicate with the VA in the future. This gap among those willing to communicate and those already communicating reveals an opportunity for better engagement through education on accessing and using SM.^16^ Few (12.6%) reported using SM to contact the VA team for menopause symptom management. However, in response the question, “How often would you use secure messaging in future for menopause symptom management?” only 29.8% said they never would, and more than three quarters (78.5%) of participants in the intervention group, and more than half (55.6%) of the comparison group mentioned that they would be willing to do so in the future. This gap again reflects an opportunity and corroborates previous studies that have shown that veterans are partial to the use of SM for sensitive topics.^16,17^

A limitation of our recruitment strategy was that only previous users of My Health*e*Vet were approached and recruited to participate in the intervention, whereas this was not the case for comparison group participants who were reached mostly *via* mail. The different recruitment strategies between groups led to the intervention group participants being almost exclusively comprising My Health*e*Vet users. This resulted in a dissimilarity in patient portal use between intervention and comparison groups. To better match the study groups, we recruited a comparison group at the same site as the intervention. Compared with the intervention group, the locally recruited comparison group demonstrated key differences in SM use and usability and willingness to use My Health*e*Vet to communicate with the VA. Not only did this recruitment strategy assist in overcoming racial and ethnic differences, but also helped account for many sociodemographic confounders associated with the digital disparities.^23^

Women report a desire for more information about menopause, menopause symptom management, and potential related health concerns.^24^ In our study, My Health*e*Vet was used to share timely information regarding menopause with patients likely to benefit, to enhance knowledge, and shared decision-making. These patients were preemptively identified using population health approaches. Menopause is a sensitive topic, and women who had concerns may have opted in for this intervention because they saw that SM provided a supplementary yet private means to get additional needed information and communicate with their physician. Previous studies have shown that women were more likely to report using SM compared with their male counterparts, as were minorities compared with nonminorities.^16,17^ This makes SM a perfect tool to approach sensitive topics while addressing gender and racial disparities as well. Information exchange through My Health*e*Vet represents an opportunity to communicate nonemergent issues and promote shared decision-making between patients with other conditions and their providers as well.

## Conclusions

Results collected from this MEANS project provide important information on the effectiveness of a patient portal intervention on knowledge and shared decision-making regarding menopause and associated conditions in women veterans. The study highlights untapped, scalable, and enormous potential for patient portals in nonurgent chronic disease management and shared decision-making.^10,25^ Our findings will provide a lens to gauge the feasibility of this framework in the existing health care system, wherein “meaningful use” of electronic health records is mandated.^12^ Because of the COVID-19 pandemic, medical care has abruptly changed to telehealth and this approach to patient education is more relevant now than ever before.

## Abbreviations Used

MEANS: My Health*e*Vet to Enable And Negotiate for Shared decision-making
SD: standard deviation
SM: secure messaging
VA: Veterans Affairs
VAHS: Veterans Affairs Healthcare Systems

## References

[1] Trudeau KJ, Ainscough JL, Trant M, Starker J, Cousineau TM Identifying the educational needs of menopausal women: A feasibility study. Womens Health Issues 2011;21:145–152 21185735 10.1016/j.whi.2010.10.001 PMC3856775

[2] Sood R, Kuhle CL, Kapoor E, et al. Association of mindfulness and stress with menopausal symptoms in midlife women. Climacteric 2019;22:377–382 30652511 10.1080/13697137.2018.1551344

[3] Katon JG, Gray KE, Gerber MR, et al. Vasomotor symptoms and quality of life among veteran and non-veteran postmenopausal women. Gerontologist 2016;56 Suppl 1:S40–S53 26220418 10.1093/geront/gnv104

[4] Avis NE, Crawford SL, Greendale G, et al. Duration of menopausal vasomotor symptoms over the menopause transition. JAMA Intern Med 2015;175:531–539 25686030 10.1001/jamainternmed.2014.8063 PMC4433164

[5] Dietz NA, Mijares-Cantrell T, Acevedo D, et al. Women veterans and menopause: Knowledge and preferences. Women Health 2018;58:898–914 28805533 10.1080/03630242.2017.1363123

[6] Resurreccion DM, Motrico E, Rigabert A, et al. Barriers for nonparticipation and dropout of women in Cardiac Rehabilitation Programs: A systematic review. J Womens Health (Larchmt) 2017;26:849–859 28388314 10.1089/jwh.2016.6249

[7] What is a patient portal? The Office of the National Coordinator for Health Information Technology (ONC) Web site. Available at: https://www.healthit.gov/faq/what-patient-portal Published 2017. Updated September 29, 2017. Accessed 11, 2018

[8] Vydra TP, Cuaresma E, Kretovics M, Bose-Brill S Diffusion and use of tethered personal health records in primary care. Perspect Health Inf Manag 2015;12:1c PMC4696089 26755897

[9] Dang S, Siddharthan K, Ruiz DI, Gomez-Orozco CA, Rodriguez R, Gomez-Marin O Evaluating an electronic health record intervention for management of heart failure among Veterans. Telemed J E Health 2018;24:1006–1013.. 29672218 10.1089/tmj.2017.0307

[10] Kuo A, Dang S Secure messaging in electronic health records and its impact on diabetes clinical outcomes: A systematic review. Telemed J E Health 2016;22:769–777 27027337 10.1089/tmj.2015.0207

[11] Davis S, Roudsari A, Raworth R, Courtney KL, MacKay L Shared decision-making using personal health record technology: A scoping review at the crossroads. J Am Med Inform Assoc 2017;24:857–866 28158573 10.1093/jamia/ocw172 PMC7651971

[12] Liebovitz D Meaningful EHR attributes for an era of accountability, transparency, shared decision making, and value assessment. J Leg Med 2013;34:43–53 23550982 10.1080/01947648.2013.768145

[13] Elwyn G, Frosch D, Thomson R, et al. Shared decision making: A model for clinical practice. J Gen Intern Med 2012;27:1361–1367 22618581 10.1007/s11606-012-2077-6 PMC3445676

[14] Dang S, Thavalathil B, Ruiz D, Gomez-Orozco C, Gomez-Marin O, Levis S A patient portal intervention for menopause knowledge and shared decision-making. J Womens Health (Larchmt) 2019;28:1614–1622 31390282 10.1089/jwh.2018.7461

[15] Elwyn G, Durand MA, Song J, et al. A three-talk model for shared decision making: Multistage consultation process. BMJ 2017;359:j4891 29109079 10.1136/bmj.j4891 PMC5683042

[16] Haun JN, Lind JD, Shimada SL, et al. Evaluating user experiences of the secure messaging tool on the Veterans Affairs' patient portal system. J Med Internet Res 2014;16:e75 24610454 10.2196/jmir.2976 PMC3961805

[17] Haun JN, Patel NR, Lind JD, Antinori N Large-scale survey findings inform patients' experiences in using secure messaging to engage in patient-provider communication and self-care management: A quantitative assessment. J Med Internet Res 2015;17:e282 26690761 10.2196/jmir.5152 PMC4704939

[18] Pew Research Center. Mobile Fact Sheet. Available at: https://www.pewresearch.org/internet/fact-sheet/mobile Published 2019. Accessed 530, 2020

[19] Bush NE, Wheeler WM Personal technology use by US military service members and veterans: An update. Telemed EHealth 2015;21:245–258 10.1089/tmj.2014.0100 25615027

[20] Irizarry T, DeVito Dabbs A, Curran CR Patient portals and patient engagement: A state of the science review. J Med Internet Res 2015;17:e148 26104044 10.2196/jmir.4255 PMC4526960

[21] Woods SS, Forsberg CW, Schwartz EC, et al. The association of patient factors, digital access, and online behavior on sustained patient portal use: A prospective cohort of enrolled users. J Med Internet Res 2017;19:e345 29042345 10.2196/jmir.7895 PMC5663951

[22] Beiter PA, Sorscher J, Henderson CJ, Talen M Do electronic medical record (EMR) demonstrations change attitudes, knowledge, skills or needs? Inform Prim Care 2008;16:221–227 19094409 10.14236/jhi.v16i3.697

[23] Ancker JS, Mauer E, Hauser D, Calman N Expanding access to high-quality plain-language patient education information through context-specific hyperlinks. AMIA Annu Symp Proc 2016;2016:277–284 28269821 PMC5333247

[24] Liao K, Hunter M Preparation for menopause: Prospective evaluation of a health education intervention for mid-aged women. Maturitas 1998;29:215–224 9699192 10.1016/s0378-5122(98)00033-4

[25] Kuo AM, Thavalathil B, Elwyn G, Nemeth Z, Dang S The promise of electronic health records to promote shared decision making: A narrative review and a look ahead. Med Decis Making 2018;38:1040–1045 30226100 10.1177/0272989X18796223

